# A New Tyrosinase Inhibitor from the Red Alga *Symphyocladia latiuscula* (Harvey) Yamada (Rhodomelaceae)

**DOI:** 10.3390/md17050295

**Published:** 2019-05-17

**Authors:** Pradeep Paudel, Aditi Wagle, Su Hui Seong, Hye Jin Park, Hyun Ah Jung, Jae Sue Choi

**Affiliations:** 1 Department of Food and Life Science, Pukyong National University, Busan 48513, Korea; phr.paudel@gmail.com (P.P.); aditiwagle05@gmail.com (A.W.); seongsuhui@naver.com (S.H.S.); 2 Department of Food Science and Nutrition, Changshin University, Gyeongsangnam-do 51352, Korea; parkhj@cs.ac.kr; 3 Department of Food Science and Human Nutrition, Chonbuk National University, Jeonju 54896, Korea

**Keywords:** *Symphyocladia latiuscula*, bromophenols, mushroom tyrosinase, B16F10, melanin

## Abstract

A marine red alga, *Symphyocladia latiuscula* (Harvey) Yamada (Rhodomelaceae), is a rich source of bromophenols with a wide array of biological activities. This study investigates the anti-tyrosinase activity of the alga. Moderate activity was demonstrated by the methanol extract of *S. latiuscula*, and subsequent column chromatography identified three bromophenols: 2,3,6-tribromo-4,5-dihydroxybenzyl methyl alcohol (**1**), 2,3,6-tribromo-4,5-dihydroxybenzyl methyl ether (**2**), and bis-(2,3,6-tribromo-4,5-dihydroxybenzyl methyl ether) (**3**). Bromophenols **1** and **3** exhibited potent competitive tyrosinase inhibitory activity against l-tyrosine substrates, with IC_50_ values of 10.78 ± 0.19 and 2.92 ± 0.04 μM, respectively. Against substrate l-3,4-dihydroxyphenylalanine (l-DOPA), compounds **1** and **3** demonstrated moderate activity, while **2** showed no observable effect. The experimental data were verified by a molecular docking study that found catalytic hydrogen and halogen interactions were responsible for the activity. In addition, compounds **1** and **3** exhibited dose-dependent inhibitory effects in melanin and intracellular tyrosinase levels in α-melanocyte-stimulating hormone (α-MSH)-induced B16F10 melanoma cells. Compounds **3** and **1** were the most effective tyrosinase inhibitors. In addition, increasing the bromine group number increased the mushroom tyrosinase inhibitory activity.

## 1. Introduction

Marine algae are widely used in a variety of cuisines [1] and are preferred by a growing number of consumers for their functional and nutraceutical properties [2,3]. The pharmaceutical industry has also developed a strong interest in algae [4,5], as have cosmetologists [6]. A major concern among the latter is hyperpigmentation, which is an abnormal darkening of the skin associated with excessive melanin production [7,8]. Although melanin is a pigment responsible for the photo-protective effect of skin, overproduction may lead to skin disorders [9]. Because tyrosinase is a rate-limiting step in melanin formation, identification of a strong tyrosinase inhibitor would be of significant value to hyperpigmentation research.

Melanogenesis describes the production of distinct melanin pigments by specialized cells called melanocytes, which are found within membrane-bound organelles known as melanosomes [10]. The embryonic precursors of melanocytes, i.e., melanoblasts, originate in neural crest cells [11]. Mature melanocytes are connected with keratinocytes through dendritic cells, which facilitate the transfer of melanin in neighboring keratinocytes cells, are responsible for skin pigmentation, and protect the skin from harmful ultraviolet radiation [12]. Melanocyte-specific markers, which are used to identify the expression of melanocyte cells in the melanogenesis process, include tyrosinase, tyrosinase-related protein 1 (TRP1), tyrosinase-related protein 2 (TRP2)/dopachrome tautomerase (DCT), pre-melanosome protein 17 (Pmel17/gp100), melan-A/melanoma antigen recognized by T-cells 1 (MART-1), and microphthalmia-associated transcription factor (MITF). In melanin synthesis, the rate-limiting step is catalyzed by tyrosinase, while TRP1 is responsible for the oxidation of 5,6-dihydroxyindole-2-carboxylic acid to a carboxylated indole quinone [13]. Moreover, TRP2/DCT enzymes catalyze the tautomerization of dopachrome into 5,6-dihydroxyindole-2-carboxylic acid, which can be detected in both melanocytes and melanoblasts [14,15]. Pmel17/gp100 is necessary for the formation of the structural matrix of stage II melanosomes [16]. MART-1, meanwhile, is specifically targeted to tumor-directed T-lymphocytes and is a probable target for immunotherapy of melanoma [17]. MITF is a major transcriptional factor in the regulation of melanocyte-specific genes encoding tyrosinase, TRP2/DCT, and MART-1 [18,19,20]. 

Melanin pigments common in humans include eumelanin (a brown-black pigment) and pheomelanin (a yellow/orange-red pigment). Melanin is produced from its precursor, l-tyrosine, through a series of enzymatic and chemical reactions. The first step of melanin biosynthesis, the oxidation of an l-tyrosine substrate, is catalyzed by the rate-limiting enzyme tyrosinase. This is followed by dopaquinone formation [21,22]. Autoxidation of dopaquinone leads to the formation of l-DOPA and dopachrome. l-DOPA acts as a co-factor in the reaction [23] and also as a substrate for tyrosinase. Eumelanin is subsequently formed through a series of oxidation reactions with dihydroxyindole and dihydroxyindole-2-carboxylic acid. Dopaquinone is condensed into cysteinyldopa or glutathionyldopa in the presence of cysteine or gluthathione, producing pheomelanin pigments. A heterogeneous pool of mixed-type melanins can also be formed through interactions between eumelanin and pheomelanin [22,24]. 

**Figure 1:**
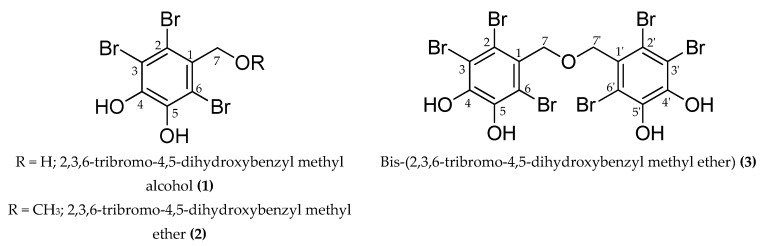
Structure of the compounds isolated from the ethyl acetate fraction of *S. latiuscula*.

Recently, marine algae have gained considerable interest in the discovery of tyrosinase inhibitors. In a study conducted to find new anti-browning and whitening agents from marine sources, Cha et al. [25] screened 43 indigenous marine algae for tyrosinase inhibitory activity and found potent tyrosinase inhibitory activity of extracts from *Endarachne binghamiae*, *Schizymenia dubyi*, *Ecklonia cava,* and *Sargassum silquastrum*. However, *Symphyocladia latiuscula* (Harvey) Yamada was not in the list of the 43 studied algae. Of the total microalgae in marine source, brown algae account for approximately 59%, followed by red algae at 40% and green algae at less than 1% [26], and phlorotannins and halogenated compounds are predominant secondary metabolites with prominent biological activities. Lately, tyrosinase inhibitory potentials of phlorotannins from marine algae have emerged with great interest [27,28,29]. Similarly, due to various advantages of halogens in drug pharmacokinetics such as lipophilicity, cell membrane solubility, membrane binding, permeation, diffusion, and half-life, chemists are focusing on the synthesis of novel tyrosinase inhibitors, taking halogenation as a basic tool [30,31,32]. However, reports on natural halogenated compounds from marine sources are limited. Therefore, this study focuses on a red alga, *Symphyocladia latiuscula,* and its constituent, bromophenols (Figure 1), for anti-tyrosinase activity.

*Symphyocladia latiuscula* (Harvey) Yamada is a member of the Rhodomelaceae family predominantly distributed along the coasts of Korea, Japan, and northern China. It is a red alga rich in bromophenols with a wide range of bioactive properties [33]. Among the compounds found in *S. latiuscula* are antioxidants [34,35], free radicals scavengers [36,37,38], peroxynitrite scavengers [39], anti-inflammatory and antibacterial [40], antifungal [41,42], antiviral [43], cytoprotective [39], and anti-diabetes [44], along with aldose reductase inhibitors [45], Taq DNA polymerase inhibitors [46], and anti-proliferators [47]. However, anti-tyrosinase activity has not yet been investigated in detail.

## 2. Results

### 2.1. Effect of Bromophenols on Tyrosinase Activity

Mushroom tyrosinase inhibitory activity of MeOH extract of *S. latiuscula* demonstrated inhibition percentages of 39.58% and 86.47% at a concentration of 1000 and 250 µg/mL for l-tyrosine and l-DOPA, respectively. This result helped determine the compounds (Figure 1) responsible for the activity in the MeOH extract of *S. latiuscula*. 

In l-tyrosine substrate, compound **3** demonstrated a potent mushroom tyrosinase inhibitory activity, with a half-maximal inhibitory concentration (IC_50_) of 2.92 ± 0.04 µM, followed by compound **1** with 10.78 ± 0.04 µM. However, compound **2** exhibited weaker inhibition, with an IC_50_ value of 113.94 ± 0.75 µM (Table 1). Weak inhibition was also exhibited by compounds in l-DOPA substrate.

### 2.2. Effect of Bromophenols on Enzyme Kinetic Inhibition

**Figure 2:**
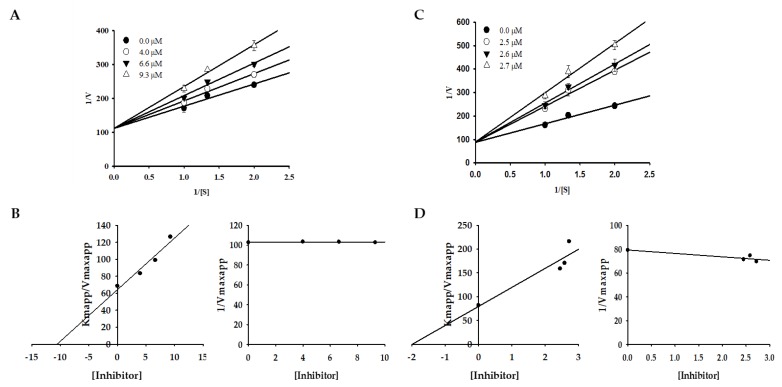
Lineweaver–Burk plots and the secondary plots of *K*_mapp_/*V*_maxapp_ and 1/*V*_maxapp_ for the inhibition of tyrosinase by compounds **1** (**A**,**B**) and **3** (**C**,**D**) in the presence of different concentrations of substrate (l-tyrosine).

Changes in *K*_m_ values were observed on a double-reciprocal Lineweaver–Burk plot, which showed that compounds **1** and **3** induced competitive-type inhibition (Table 1, Figure 2). Furthermore, the secondary replot of *K*_mapp_/*V*_maxapp_ and 1/*V*_maxapp_ versus compounds **1** and **3** was used to determine the binding constant of the inhibitor for free enzymes (*K*_ic_). The *K*_ic_ (≈*K*_i_) values for compounds **1** and **3** were 10.59 and 1.98 µM. 

### 2.3. Molecular Docking Simulation on Tyrosinase Inhibition

**Figure 3:**
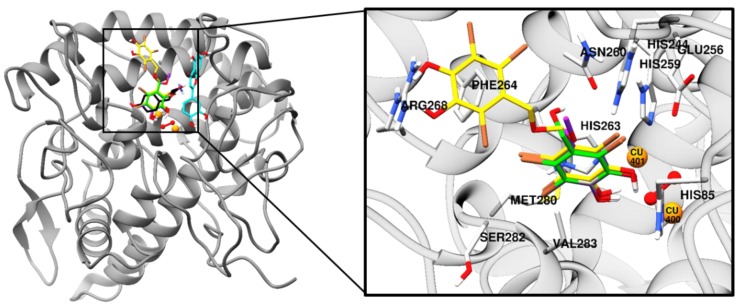
Molecular docking results of bromophenol compounds from *S. latiuscula* in the active site of oxy-form *Agaricus bisporus* tyrosinase (2Y9X) along with reference ligands. The chemical structure of compounds **1**, **2**, **3,** l-tyrosine, and luteolin are shown in green, purple, yellow, black, and cyan sticks, respectively. Bromine, oxygen, and nitrogen atoms are shown in brown, red, and blue, respectively. Copper and peroxide ions are shown in orange and red spheres, respectively.

**Figure 4:**
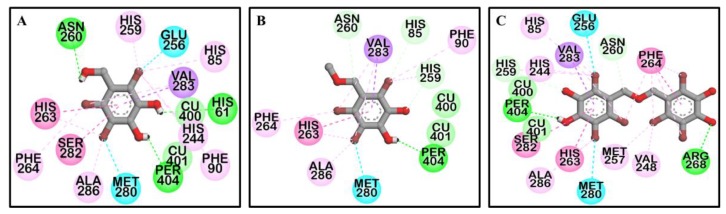
Molecular docking results of bromo-compounds **1** (**A**), **2** (**B**), and **3** (**C**) in the catalytic site of oxy-form *Agaricus bisporus* tyrosinase (2Y9X).

To predict the binding site of the active compounds, a molecular docking simulation was performed. The binding energy, the number of hydrogen bonds, and the hydrogen-bond interaction, along with other interaction residues of the compounds and the reference compounds l-tyrosine (competitive inhibitor) and luteolin (allosteric inhibitor) are summarized in Table 2 and Figure 3 and Figure 4. The most potent bromophenol, compound **3**, formed two hydrogen-bond interactions with Arg268 and peroxide ions (Per404) between the two copper ions. Furthermore, the complex was stabilized by His259, Asn260, Glu256, Met280, Val283, His263, Phe264, Ser282, Ala286, Val248, Met257, Val283, His85, His244, His259, His263, and Phe264 interacting residues (Figure 4C). Similarly, together with the three-hydrogen-bond interaction with Per404, Asn260, and His61, the compound **1**‒tyrosinase complex was stabilized by Glu256, Met280, Val283, His263, Ala286, Val283, His85, Phe90, His244, His259, His263, and Phe264, as shown in Figure 4A. Likewise, only one hydrogen bond with peroxide ions was found with compound **2** (Figure 4B). The binding energies of −6.19, −6.29, and −7.81 kcal/mol were consumed by compounds **1**, **2**, and **3**, respectively.

### 2.4. Effect of Bromophenols on Cell Viability of B16F10 Cells

**Figure 5:**
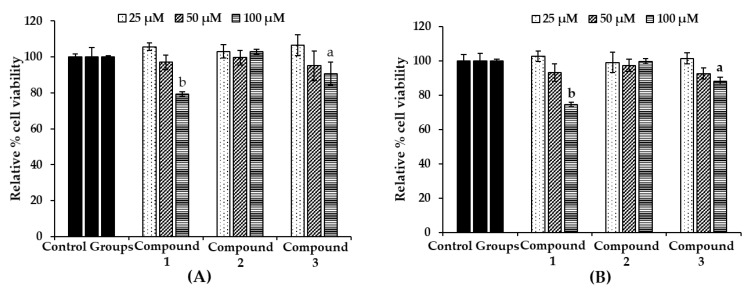
Effect of bromophenols **1**–**3** on cell viability in B16F10 cells (**A**) and co-treatment with 5 μM α-MSH (**B**). Cell viability was determined using the 3-(4,5-dimethylthiazol-2-yl)-2,5-diphenyl tetrazolium bromide (MTT) method. Cells were pretreated with the indicated concentrations (25, 50, and 100 μM) of test compounds for 48 h. Data shown represent mean ± SD of triplicate experiments. ^a^ *p* < 0.05 and ^b^ *p* < 0.01 indicates significant differences from the control group.

To evaluate the toxicity of the bromophenols in B16F10 melanoma cells, cell viability upon bromophenol treatment only (Figure 5A) and/or co-treatment with 5 µM α-MSH for 48 h (Figure 5B) was determined using a 3-(4,5-dimethylthiazol-2-yl)-2,5-diphenyltetrazolium bromide (MTT) assay. The B16F10 melanoma cells were treated at a concentration of 25–100 µM of isolated bromophenols for 48 h. Bromophenol **2** showed no toxicity up to a concentration of 100 µM (approximately 100% viable cells). However, compounds **1** and **3** displayed significant toxicity (74.6% and 86.9% viable cells, respectively). No toxicity was observed up to 50 µM concentration for all compounds. Consequently, a 25 µM sample concentration was used for further experiments.

### 2.5. Effect of Bromophenols on Melanin Content and Intracellular Tyrosinase Activity in B16F10 Cells

**Figure 6:**
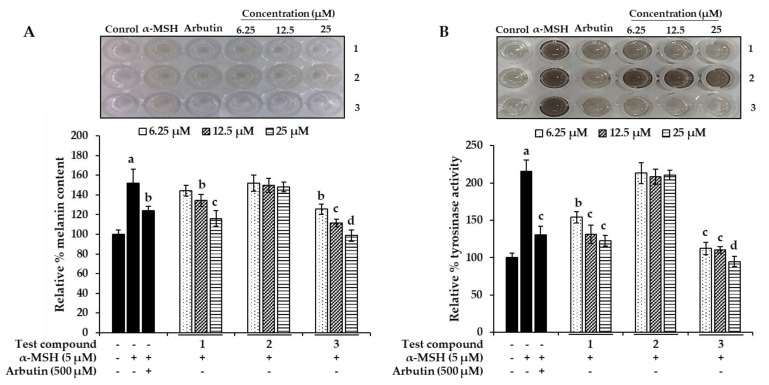
Effect of bromophenols **1**–**3** on extracellular melanin content (**A**) and cellular tyrosinase activity (**B**) in α-MSH-stimulated B16F10 cells. Cells were pretreated with the indicated concentrations (6.25, 12.5, and 25 μM) of bromophenols **1**–**3** for 1 h followed by exposure to α-MSH (5.0 μM) for 48 h in the presence or the absence of test bromophenols. Arbutin (500 μM) was used as a positive control. Values represent the mean ± SD of triplicate experiments. ^a^ *p* < 0.01 indicates significant differences from the control group; ^b^ *p* < 0.05, ^c^ *p* < 0.01 and ^d^ *p* < 0.001 indicate significant differences from the α-MSH treated group.

Melanin and intracellular tyrosinase (TYR) contents were measured after pretreatment of B16F10 melanoma cells with different concentrations (6.25, 12.5, and 25 µM) of the three bromophenols for 1 h, followed by 48 h of α-MSH treatment. After stimulating B16F10 cells with 5 µM α-MSH for 48 h, the melanin content rose to 151.72% (Figure 6A). However, treatment of bromophenols **1** and **3** reduced the melanin content in a dose-dependent manner. At a 25 µM concentration, compounds **1** and **3** reduced the melanin content to 115.94% and 98.68%, respectively. Arbutin at a 500 µM concentration reduced the content to 124.06%. In parallel with the enzyme assay, compound **2** showed no significant reduction in the melanin content. Because cellular tyrosinase enhances melanin overproduction, reduction of tyrosinase activity is an efficient strategy for the development of anti-melanogenic agents. A l-DOPA oxidation protocol was designed to examine the inhibitory activity of isolated bromophenols against tyrosinase in α-MSH-induced B16F10 melanoma cells, because l-tyrosine and l-DOPA are sequentially generated substrates that regulate melanogenesis and modulate melanocyte function through overlapping substrates. After 48 h of sample treatment, intracellular tyrosinase activity was measured. As shown in Figure 6B, with treatments **1** and **3**, intracellular tyrosinase activity decreased in a dose-dependent manner compared with controls. The level of intracellular tyrosinase after α-MSH treatment was 215.73%, which was reduced to 122.64% and 94.81% after treatment with 25 µM concentration of **1** and **3**, respectively. The activities of **1** and **3** were better than that of 500 µM arbutin, which reduced intracellular tyrosinase levels to 130.75%. As with melanin content, compound **2** did not show a significant reduction in intracellular tyrosinase level at tested concentrations.

### 2.6. Effect of Bromophenols on Tyrosinase Expression

**Figure 7:**
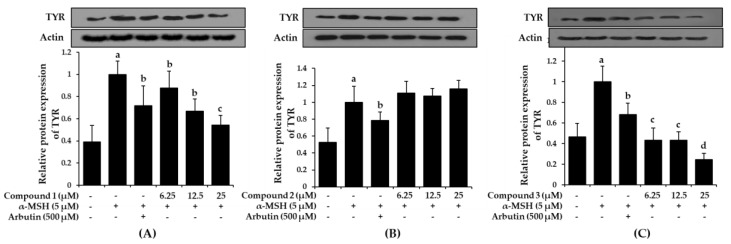
Effects of bromophenol **1** (**A**), **2** (**B**), and **3** (**C**) on cellular tyrosinase protein expression levels in α-MSH-stimulated B16F10 cells. Western blotting was performed, and protein band intensities were quantified by densitometric analysis. Upper panels display representative blots. Graphs under each bands represent the relative band density for tyrosinase (TYR) normalized to β-actin. Values represent the mean ± SD of three independent experiments; ^a^ *p* < 0.001 indicates significant differences from the control group; ^b^ *p* < 0.05, ^c^ *p* < 0.01 and ^d^ *p* < 0.001 indicate significant differences from the α-MSH treated group.

Increased tyrosinase activity enhances melanogenesis and hyperpigmentation, implying that downregulation of tyrosinase activity would theoretically represent an anti-pigmenting property. To evaluate the effect of bromophenols **1**-**3** on tyrosinase expression, the B16F10 cells were pre-treated with the bromophenols before stimulation with α-MSH, and the tyrosinase protein levels were examined using western blot analysis. As shown in Figure 7, treatment with compounds **1** and **3** at concentrations of 6.25, 12.5, and 25 μM for 48 h inhibited α-MSH-induced accumulation of tyrosinase proteins in a concentration-dependent manner. 

Compared with the normal control group, which had low levels of tyrosinase expression, the α-MSH-treated group demonstrated intensified tyrosinase expression levels. However, pre-treatment with arbutin and/or bromophenols **1** and **3** significantly reduced the expression levels. Compound **1** at 25 μM and compound **3** at 6.25 μM were more effective at downregulating tyrosinase expression than arbutin. Compound **2** did not reduce expression up to 25 μM.

## 3. Discussion

Use of cosmetics can lead to a variety of adverse effects and allergies in users sensitive to certain chemicals and other ingredients. To avoid unnecessary interactions and to reduce adverse effects of synthetic and semi-synthetic cosmetics, consumer preferences are shifting toward natural products. Marine species are an abundant source of chemical and bioactive compounds due to the variety of biological activities associated with marine organisms [48]. Many compounds derived from marine species with possible applications in the nutraceutical and cosmetics fields remain unexamined [49,50].

To meet the demand for new compounds that exhibit potent anti-tyrosinase activity, a preliminary screening of the methanolic extract of *S. latiuscula* was performed. The study revealed that a MeOH extract was associated with inhibition rates of 39.58% and 86.47% at concentrations of 1000 and 250 µg/mL for l-tyrosine and l-DOPA, respectively. A study performed by Seo and Yoo [51] reported inhibition rates of 84% and 60% against MeOH extract and a mixture of acetone and methylene chloride, respectively, using l-tyrosine as a substrate. In contrast, no inhibition was observed at a concentration of 500 µg/mL [27]. The possible reasons for the discrepancies among these experiments include the difference in concentrations of enzyme and substrate, incubation time, and environmental conditions. Based on the results, *S. latiuscula* extract was subjected to bio-assay guided fractionation, which yielded 2,3,6-tribromo-4,5-dihydroxybenzyl methyl alcohol (**1**), 2,3,6-tribromo-4,5-dihydroxybenzyl methyl ether (**2**), and bis-(2,3,6-tribromo-4,5-dihydroxybenzyl methyl ether) (**3**). For the first time, a mushroom tyrosinase inhibitory assay with an l-tyrosine substrate for the isolated compounds revealed compound **3** to be approximately twice and 23 times more potent than compounds **1** and **2**, respectively (Table 1). A similar tendency was observed for the l-DOPA substrate but with only moderate potency. This result highlights the dimeric form of compound **2**, specifically how the *O*-linkage of the 2,3,6-tribromo-4,5-dihydroxyl methyl ether element enhances mushroom tyrosinase inhibition. Moreover, an increased number for bromophenol moiety contributes to increased inhibition toward tyrosinase. The results were further verified by the observations of B16F10 melanoma cells, which had concentration-dependent inhibitory effects on melanin content and tyrosinase activity for compounds **1** and **3**. A similar tendency for reduction of tyrosinase expression levels by **1** and **3** was observed in a western blot analysis.

To shed light on the inhibition mode against tyrosinase, kinetic analysis for active compounds **1** and **3** using an l-tyrosine substrate was performed, supplemented by a molecular docking study. A double-reciprocal Lineweaver‒Burk plot of compounds **1** and **3** for the oxidation of different concentrations of l-tyrosine demonstrated competitive inhibition of both compounds, as *K_m_* changed while *V_max_* was constant. Graphing 1/V versus 1/[S] produced a family of straight lines with diverse slopes, although the lines intersected on the *Y*-axis. The results showed that compounds **1** and **3** could only bind with free enzymes. An inhibition constant for the inhibitor binding with the free enzyme (*K_ic_*) was obtained from the secondary plot as 10.59 and 1.98 µM.

To predict binding affinity, activity of the small molecules, and the interaction of the molecules, and to determine the optimal orientation of the protein–ligand complex with minimum energy, molecular docking tools can be applied. This helps reveal the underlying mechanism of the compound responsible for a particular bioactivity. A simulated docking study of the binding of the compounds to the catalytic site of tyrosinase (Figure 3 and Figure 4) provided evidence of the competitive inhibition, as shown by a kinetic analysis (Figure 3). An H-bond interaction was formed by compound **1** as the fourth and fifth hydroxyl group interacted with the peroxide ion located between the two copper ions and His61, respectively, and the seventh hydroxyl group interacted with an Asn260 amino-acid residue. Moreover, the O-atom at the carboxylic moiety of Glu256 and Met280 interacted with the third- and the sixth-position bromine atoms. The complex was stabilized by hydrophobic interactions with Val283, His263, and Ser282 and halogen interactions with Ala286, Val283, His85, Phe90, His244, His259, His263, and Phe264 residues (Figure 4A). Although similar interactions were observed for compound **3**, the strong H-bond interaction between Arg268 residue and the 4′-OH group of active compound **3** helped stabilize the compound **3**‒tyrosinase complex [52]. Because of the increase in the bromine moiety number, there was an increase in halogen interactions assisting the potent anti-tyrosinase activity (Figure 4C). A similar result was seen in tyrosine phosphatase 1B and α-glucosidase inhibition [44]. Although compound **2** lacked the H-bond interaction with the other amino-acid residues, a peroxide ion between the two copper atoms can explain its weak tyrosinase inhibitory activity (Figure 4B). The lower the value of binding energy shown by the compounds was, the more stable the complex formed between the ligand and targeted protein was. Binding energies of compounds **1**–**3** to tyrosinase was comparable. However, compound **2** showed ineffective anti-melanogenic activity. The probable reason for this discrepancy might be that, despite the fact that compound **2** was involved in van der Waals interactions with copper ions (Cu401 and 400) and H-bond interactions with a peroxide ion (Per404) at the active site, the complex was not stable. For **1** and **3**, H-bond interactions with Asn260 and Arg268 and hydrophobic interaction with Ser282 most probably stabilized the complex, and these interactions were not observed for **2**. However, this should be confirmed through molecular dynamic simulation. Moreover, as the present study reports just three compounds, the structural-activity relationship study might be incomplete. Therefore, in depth study of the structural-activity relationship with a larger number of bromophenols is warranted. The results confirm the strong activity exhibited by the compounds isolated from *S. latiuscula,* making the alga a possible source for depigmenting agents in cosmetology.

## 4. Materials and Methods 

### 4.1. Chemicals and Reagents

l-Tyrosine, l-3,4-dihydroxyphenylalanine (l-DOPA), arbutin, mushroom tyrosinase enzyme (EC 1.14.18.1), and α-melanocyte-stimulating hormone (α-MSH) were obtained from Sigma Aldrich (St. Louis, MO, USA). Dulbecco’s modified Eagle’s medium (DMEM), fetal bovine serum (FBS), and penicillin-streptomycin were purchased from Gibco-BRL Life Technologies (Grand Island, NY, USA). Primary tyrosinase (TYR) antibodies, β-actin, and horseradish peroxidase-conjugated secondary antibodies were obtained from Santa Cruz Biotechnology Inc. (Santa Cruz, CA, USA). Dipotassium phosphate and monopotassium phosphate were obtained from Junsei Chemical Co. Ltd. (Tokyo, Japan) and Yakuri Pure Chemicals Co. Ltd. (Osaka, Japan), respectively. All other reagents and solvents were purchased from E. Merck, Fluka, and Sigma-Aldrich unless otherwise stated.

### 4.2. Algal Material

Leafy thalli of *S. latiuscula* (Harvey) Yamada collected from Cheongsapo, Busan, Korea, in January 2016 were authenticated by an algologist, Doctor K. W. Nam, at the Department of Marine Biology, Pukyong National University. A voucher specimen (No. 20160140) was deposited in the laboratory of professor J. S. Choi, Pukyong National University.

### 4.3. Extraction, Fractionation, and Isolation

Clean and dried leafy thalli of *S. latiuscula* (Harvey) Yamada (700 g) was extracted in methanol (MeOH) three times successively for 3 h at a time (5 L × 3 times) under reflux, followed by concentration until dry in vacuo at 40 °C to obtain 207.38 g of MeOH extract. The resulting MeOH extract was successively partitioned with different solvent soluble fractions. Details on partition, isolation, and identification of compounds are reported in Paudel et al. [44].

### 4.4. Mushroom Tyrosinase Inhibitory Assay

The inhibitory activity of mushroom tyrosinase was carried out using a spectrophotometric method described in [27] with slight modifications. In brief, 10 µL of samples/positive control (kojic acid) together with 50 mM of phosphate buffer (pH 6.5), 1 mM of substrate (l-tyrosine/l-DOPA) solution, and distilled water were mixed at a ratio of 10:10:9 in a 96-well plate. Then, 20 µL of tyrosinase was added and incubated at room temperature for between 10 and 30 minutes. Absorbance was measured at 490 nm after incubation.

### 4.5. Kinetic Study Against Mushroom Tyrosinase

The inhibition type was determined using a Lineweaver–Burk plot [53]. The apparent slope (*K*_mapp_/*V*_maxapp_) and intercept (1/*V*_maxapp_) versus different inhibitor concentrations was used to determine the inhibition constant (*K*_i_) [54]. Different kinetic parameters were obtained for different concentrations of the substrate (0.5, 0.75, and 1 mM) and inhibitors (compound **1**—0.0, 4.0, 6.6, and 9.3 µM; compound **3**—0, 2.5, 2.6, and 2.7 µM). Sigmaplot version 12.0 (SPSS Inc., Chicago, IL, USA) was employed to generate the plots.

### 4.6. Molecular Docking Simulation of Mushroom Tyrosinase

Molecular docking analysis was carried out using the procedure described [55] in AutoDock 4.2. Structures for l-tyrosine (6057) and luteolin (5280445) were obtained from the PubChem compound database. The three-dimensional (3D) structures of the bromophenol compounds were generated by Marvin Sketch (v17.1.30, ChemAxon, Budapest, Hungary). Docking results were visualized and analyzed using PyMOL (v1.7.4, Schrödinger, LLC, Cambridge, MA) and Discovery Studio (v16.1, Accelrys, San Diego, CA, USA).

### 4.7. Cell Culture and Viability Assay

B16F10 mouse melanoma cells obtained from American Type Culture Collection (Manassas, VA, USA) were maintained in 10% FBS in high-glucose DMEM containing 100 U/mL penicillin and 100 μg/mL streptomycin at 37 °C in a humidified atmosphere with 5% CO_2_. Cytotoxicity was evaluated using a 3-(4,5-dimethylthiazol-2-yl)-2,5-diphenyltetrazolium bromide (MTT) assay. After 95% of confluence, B16F10 cells were seeded in a 96-well plate at 10,000 cells/well and incubated for 24 h in DMEM supplemented with 10% FBS. The cells were then fed fresh serum-free DMEM containing different concentrations (25 to 100 μM) of the test compounds and incubated for 48 h. After that, the cells were incubated with 100 μL of MTT at a concentration of 0.5 μg/mL in phosphate-buffered saline (PBS) for 2 h. Absorbance was measured at 490 nm by a microplate reader (Promega Corporation Instrument, USA) after 2 h of incubation. The viability of cells at 48 h upon co-treatment with α-MSH was evaluated by treating cells with 5 μM α-MSH 1 h after the treatment with test compounds.

### 4.8. Melanin Content Assay

Intracellular melanin content was determined according to a previously reported procedure [56] with minor modifications. Briefly, B16F10 cells were seeded in a 24-well plate at 2 × 10,000 cells/well and incubated for 24 h in DMEM containing 10% FBS. Cells were pretreated with 6.25, 12.5, and 25 μM test compounds for 1 h and stimulated with 5 μM α-MSH for 48 h. Arbutin (500 μM) was used as a positive control. The cells were washed with PBS and dissolved in 1 N NaOH containing 10% DMSO by boiling at 80 °C for 30 min. The cell lysates were centrifuged at 14,000 rpm for 20 min, and absorbance of the supernatant was measured at 405 nm. Melanin content was determined by normalizing the absorbance with total protein content.

### 4.9. Cellular Tyrosinase Assay

Intracellular tyrosinase inhibitory activity was evaluated by measuring the rate of oxidation of l-DOPA [57]. Briefly, 2 × 10,000 cells/well were seeded and incubated for 24 h in a 6-well plate and treated with various concentrations of bromophenols (6.25, 12.5, and 25 μM) or arbutin (500 µM) for 1 h, then stimulated with α-MSH (5 µM) for 48 h. The cells were washed with PBS and lysed with a radio immunoprecipitation assay buffer. The cell lysates were kept in a deep freezer (−80 °C) for 1 h. After defrosting the cell lysates, the cellular extracts were purified by centrifugation at 14,000 rpm for 20 min at 4 °C, and the supernatant was used as cellular tyrosinase solution. A total of 80 µL of supernatant and 20 µL of l-DOPA (2 mg/mL) were added to a 96-well plate and incubated at 37 °C for 45 min. Dopachrome formation was then measured spectrophotometrically at 450 nm using a microplate spectrophotometer (Molecular Devices). Intracellular tyrosinase activity was calculated as the percentage of control.

### 4.10. Determination of Tyrosinase Protein Levels via Western Blotting

After stimulating B16F10 cells with α-MSH in the presence or the absence of test bromophenols and arbutin for the indicated times, the cells were washed with ice-cold PBS and harvested using a cell scraper. The cell suspensions were centrifuged at 16,000 × *g* for 5 min, and the cell pellets were lysed in a lysis buffer and incubated on ice for 10 min. The cell lysates were centrifuged at 16,000 × *g* for 10 min, and the supernatant (total protein) was normalized using a Bradford protein assay kit. Aliquots of protein were resolved by sodium dodecyl sulfate-polyacrylamide gel electrophoresis and transferred onto a nitrocellulose membrane. The membrane was blocked with 10% nonfat milk (*w*/*v*) in tris-buffered saline with tween (TBST) (0.1 M Tris-HCl, pH 7.5, 1.5 M NaCl, 1% Tween20) for 2 h and incubated for approximately 24 h with TYR primary antibody. The immunoblots were then incubated with appropriate secondary antibodies for 2 h and detected using an enhanced chemiluminescence detection kit.

### 4.11. Statistical Analysis

One-way ANOVA and a Student’s *t*-test (Systat Inc., Evanston, IL, USA) were used to determine statistical significance. Values of *p* < 0.05, 0.01, 0.001, and 0.0001 were considered significant. All results are presented as the mean ± SD of triplicate experiments.

## 5. Conclusions

The present study revealed that, among three bromophenols isolated from the marine alga *S. latiuscula*, compound **3** followed by **1** exhibited potent competitive tyrosinase inhibition against l-tyrosine substrate. A molecular docking simulation of the catalytic residue revealed the underlying inhibitory mechanism. In addition, compounds **1** and **3** inhibited cellular tyrosinase activity, decreased tyrosinase protein expression levels, and reduced the melanin content in α-MSH-treated B16F10 melanoma cells in a concentration-dependent manner. These results suggest that the increased number of bromine groups in the compound is associated with significant mushroom tyrosinase inhibitory activity. Overall, the strong tyrosinase inhibitory activity exhibited by the bromophenols isolated from *S. latiuscula* makes the alga a possible source for depigmenting agents in cosmetology.

## Figures and Tables

**Table 1:** Mushroom tyrosinase inhibitory potential of bromo-compounds from *S. latiuscula*.

Compounds	IC_50_ (µM) ^a^ (*n* = 3)		l-Tyrosine	
	l-Tyrosine	l-DOPA	*K*_i_ Value (µM) ^b^	Inhibition Type ^c^
**1**	10.78 ± 0.19 ^h^	270.53 ± 2.04 ^f^	10.59	Competitive
**2**	113.94 ± 0.75 ^g^	>300	- ^d^	- ^d^
**3**	2.92 ± 0.04 ^i^	110.91 ± 4.95 ^g^	1.98	Competitive
Kojic acid ^e^	3.17 ± 0.07 ^i^	3.07 ± 0.04 ^h^	- ^d^	- ^d^
Arbutin ^e^	172.82 ± 4.71 ^f^	>300	- ^d^	- ^d^

^a^ The 50% inhibitory concentration (µM) expressed as mean ± SD of triplicate experiments; ^b^ The inhibition constant (*K*_i_) was determined from secondary plots; ^c^ Inhibition type determined from Lineweaver-Burk plots; ^d^ Not determined; ^e^ Used as reference drug; ^f–i^ Means with different letters are significantly different with Duncan’s test at *p* < 0.05.

**Table 2:** Binding energy and interacting residues of bromo-compounds from *S. latiuscula* against oxy-form *Agaricus bisporus* tyrosinase (2Y9X).

Compounds	Binding Energy (kcal/mol)	No. of H-Bonds	H-Bond Interactions	Other Interacting Residues
**1**	‒6.19	3	Asn260, His61, and Per404 (O–H bond)	Glu256 and Met280 (O-Br bond), Val283 (Pi-Sigma), His263 (Pi-Pi Stacked), Ser282 (Amide-Pi Stacked), Ala286 and Val283 (Alkyl-Br), His85, Phe90, His244, His259, His263, and Phe264 (Pi-Br), Cu401, and 400 (van der Waals)
**2**	‒6.29	1	Per404 (O–H bond)	Met280 (O-Br bond), Val283 (Pi-Sigma), His263 (Pi-Pi Stacked), Ala286 and Val283 (Alkyl-Br), His85, Phe90, His259, His263, and Phe264 (Pi-Br), Cu401, and 400 (van der Waals)
**3**	‒7.81	2	Arg268 and Per404 (O–H bond)	His259 and Asn260 (C-O bond), Glu256 and Met280 (O-Br bond), Val283 (Pi-Sigma), His263 (Pi-Pi Stacked), Phe264 (Pi-Pi T-shaped), Ser282 (Amide-Pi Stacked), Ala286, Val248, Met257, and Val283 (Alkyl-Br), His85, His244, His259, His263, and Phe264 (Pi-Br), Cu401, and 400 (van der Waals)
l-Tyrosine ^a^	‒6.31	5	His244, Asn260, and Met280 (O–H bond), Glu256 (Salt-bridge)	Ala286 (Pi-Alkyl), Val283(Pi-Sigma), His263 (Pi-Pi Stacked), Cu401, and 400, Per402 (van der Waals)
Luteolin ^a^	‒5.77	4	Cys83, Gly245, Ala246, and Val248 (O–H bond)	Val248 (Pi-Alkyl), His85 (Pi-Sigma), Glu322 (Pi-Anion)

^a^l-tyrosine and luteolin were used as reference catalytic and allosteric inhibitor, respectively.
